# Regional Targeting of Bladder and Urethra Afferents in the Lumbosacral Spinal Cord of Male and Female Rats: A Multiscale Analysis

**DOI:** 10.1523/ENEURO.0364-21.2021

**Published:** 2021-12-10

**Authors:** J. P. Fuller-Jackson, P. B. Osborne, J. R. Keast

**Affiliations:** Department of Anatomy and Physiology, University of Melbourne, Parkville, Victoria 3010, Australia

**Keywords:** continence, micturition, parasympathetic, preganglionic, sacral spinal cord, visceral afferent

## Abstract

Sensorimotor circuits of the lumbosacral spinal cord are required for lower urinary tract (LUT) regulation as well as being engaged in pelvic pain states. To date, no molecular markers have been identified to enable specific visualization of LUT afferents, which are embedded within spinal cord segments that also subserve somatic functions. Moreover, previous studies have not fully investigated the patterning within or across spinal segments, compared afferent innervation of the bladder and urethra, or explored possible structural sex differences in these pathways. We have addressed these questions in adult Sprague Dawley rats, using intramural microinjection of the tract tracer, B subunit of cholera toxin (CTB). Afferent distribution was analyzed within individual sections and 3D reconstructions from sections across four spinal cord segments (L5-S2), and in cleared intact spinal cord viewed with light sheet microscopy. Simultaneous mapping of preganglionic neurons showed their location throughout S1 but restricted to the caudal half of L6. Afferents from both LUT regions extended from L5 to S2, even where preganglionic motor pathways were absent. In L6 and S1, most afferents were associated with the sacral preganglionic nucleus (SPN) and sacral dorsal commissural nucleus (SDCom), with very few in the superficial laminae of the dorsal horn. Spinal innervation patterns by bladder and urethra afferents were remarkably similar, likewise the patterning in male and female rats. In conclusion, microscale to macroscale mapping has identified distinct features of LUT afferent projections to the lumbosacral cord and provided a new anatomic approach for future studies on plasticity, injury responses, and modeling of these pathways.

## Significance Statement

In this multiscale neuroanatomical study, we have developed novel maps of bladder and urethra afferent pathways in the spinal cord of male and female rats. Afferents visualized by transganglionic tracing were registered to anatomic landmarks including motoneuron pools and preganglionic autonomic neurons, enabling comparisons across animals and specific regions of the cord. We integrated data from 3D reconstructions of sections, light sheet microscopy of cleared, contiguous spinal segments and confocal microscopy to assess connectivity at microscale. In addition to providing new understanding of lumbosacral visceral circuitry, this dataset provides the foundation for future studies on plasticity and regenerative capacity of these circuits, changes during the life cycle and parameters for modeling spinal circuits relevant to lower urinary tract (LUT) function.

## Introduction

Healthy urinary voiding and continence require coordinated activity of the visceral and somatic nervous systems to ensure that autonomic control of the bladder and urethra is fully integrated with the voluntary controlled striated muscle of the urethral rhabdosphincter (Fowler et al., 2008; Holstege and Collewijn, 2009; Roy and Green, 2019). These behaviors depend on afferent signals that encode information about the physiological state of the lower urinary tract (LUT), such as bladder fullness, urine flow through the urethra, and tissue health (e.g., inflammation or infection). This has led to the sensory pathways being targeted for development of neuromodulation therapies that influence electrical activity of the peripheral nerve pathways and/or spinal circuits (de Groat and Tai, 2015; Shahriari et al., 2020).

To assist further development, it is important to have a more complete understanding of the 3D trajectories of bladder and urethra afferents within the spinal cord, and their connectivity with regions that influence autonomic or nociceptive function. In rats and mice, the majority of LUT afferent pathways originate from L6-S1 dorsal root ganglia (DRGs) and signal to equivalent segments of spinal cord (Applebaum et al., 1980; Keast and de Groat, 1992; Vera and Nadelhaft, 1992; Wang et al., 1998; Fowler et al., 2008). However, these spinal segments are not dedicated to LUT function but also contain sensory, motor, and integrative elements related to other pelvic visceral (lower bowel, reproductive tract) or somatic functions (hindlimb, pelvic floor, tail) (Inyama et al., 1986). Therefore, understanding the specific projections of LUT afferents requires visual discrimination of their distribution within these segments.

The aims of this study were to comprehensively map bladder and urethra afferents in the lumbosacral cord of adult male and female rats. No unique molecular markers are currently available to selectively visualize bladder or urethral afferents, although recent transcriptome analyses of mouse DRGs have defined several clusters of genes that show high expression in bladder afferents and several differences in expression level from colonic afferents (Meerschaert et al., 2020). Neural tract tracing, therefore, continues to be the most robust approach for visualizing specific nerve-organ connectivity. Visualization of spinal terminals of organ-projecting afferents requires tracers that are “transganglionic,” i.e., are transported from the organ beyond the DRG. We therefore chose the intramural injection approach, using the non-toxic B subunit of cholera toxin (CTB) as the transganglionic tracer. Studies of colonic afferents have demonstrated that CTB labels both myelinated and unmyelinated afferent classes (Christianson et al., 2006), in contrast to somatic pathways where CTB is reported to preferentially label myelinated afferents (Robertson and Grant, 1985; Shehab and Hughes, 2011) and damaged unmyelinated afferents (Tong et al., 1999; Shehab et al., 2003). We also chose immunohistochemical amplification (anti-CTB antibodies) to maximize the visibility of CTB tracer, providing greater sensitivity for mapping axons than direct visualization of fluorescent conjugates of CTB.

Our study has been designed to aggregate spatial data on these LUT afferent pathways from the microscale to macroscale. This includes confocal microscopy of LUT afferent fields within individual spinal cord sections, using these sections to reconstruct four contiguous segments of spinal cord (L5-S2) to detect regional patterning of afferent projections, and light sheet microscopy of multisegmental samples of cleared spinal cord. A critical element of our study design was the parallel collection of data on preganglionic neurons, an adjacent interneuron population, and specific pools of motoneurons; these groups of neurons were registered to spinal segment boundaries, as identified by entry points of spinal roots. Registering to these anatomic landmarks enabled us to not only detect heterogeneous distribution of afferents across an individual spinal cord segment but also to compare afferent patterning between animals. These outcomes form a fundamental dataset that can be used to build and interpret new studies on plasticity and regenerative capacity of these circuits, in addition to providing parameters for modeling spinal circuits relevant to LUT function.

## Materials and Methods

### Animals

A total of 19 male and seven female young adult Sprague Dawley rats (8–10 weeks of age; males 300–350 g, females 200–250 g) were used for this study. Stage of the estrus cycle was not recorded for the female rats. The Animal Ethics Committee of the University of Melbourne approved all animal procedures; all procedures complied with the Australian Code for the Care and Use of Animals for Scientific Purposes (National Health and Medical Research Council of Australia). Rats were housed in groups of at least two under a 12/12 h light/dark cycle with *ad libitum* food and water.

### Intramural injection of neural tracer into LUT organs

Microinjection of CTB (low salt formulation; List Biological Labs) was performed into the wall of selected regions of the LUT under isoflurane anesthesia (3% in oxygen for induction, 1–2% for maintenance), as described in detail previously (Keast and Osborne, 2019a). For each rat, CTB was injected into one LUT organ, either the bladder or the urethra. In brief, the organ of interest was accessed via a midline ventral incision, and injections of tracer made using a Hamilton Neuros Syringe attached to a 33-G needle (Hamilton). For the bladder body, a maximum volume of 3.5 μl was injected, distributed across seven sites (midline and bilaterally on both dorsal and ventral aspects, plus a single injection near the apex). For the proximal urethra, a single injection (maximum 0.5 μl) was made, following gentle blunt dissection to separate any adjacent nearby organs. Following each injection, the needle remained in place for a minimum of 5 s, then the site swabbed and rinsed with saline to minimize tracer leakage onto adjacent organs. The abdominal wall and skin were closed with sutures and surgical staples, respectively. Buprenorphine (Clifford Hallam Healthcare; 0.05 mg/kg, at the time of surgery and 8 h postsurgery) and meloxicam (Troy Laboratories; 1 mg/kg, 8 and 24 h postsurgery) were administered subcutaneously as postoperative analgesia. No adverse consequences were observed postsurgery.

### Tissue collection

We first undertook a qualitative pilot study to compare the density and distribution of CTB labeling of LUT afferents in the spinal cord after 4 or 7 d transport time (*n* = 3 rats per group, either bladder or urethra injection). This demonstrated similar outcomes, so our primary quantitative study was performed using the 4-d transport period. To collect tissue after tracer injection, animals were anesthetized (ketamine 100 mg/kg and xylazine 10 mg/kg, i.p.) and fixed by intracardiac infusion perfusion, following a detailed published protocol (Keast and Osborne, 2019b). This comprised initial perfusion with 1% sodium nitrite and 5000 IU/ml heparin in 0.9% saline for 3 min, followed by 4% paraformaldehyde in 0.1 m phosphate buffer (pH 7.4) for 8–10 min. Following perfusion, the lumbosacral spinal cord (L5-S2) and L6 DRG were removed and postfixed for 1 h in the same fixative. The tissues were then washed three times in 0.1 m PBS, pH 7.2, for 1 h each wash and stored at 4°C until use.

### Classification of DRG neurons labeled by LUT injection of CTB

To determine the uptake of CTB by primary classes of LUT afferents, L6 DRGs were analyzed from 6 male rats (three rats for bladder body injection and a different group of three rats for urethra injection). Ganglia were cryoprotected overnight in 0.1 m PBS containing 30% sucrose before embedding in optimal cutting temperature (OCT) compound (Sakura Finetek). Cryosections (14 μm) were collected on gelatinized glass slides, dried and washed in 0.1 m PBS before being incubated in 0.1 m PBS containing 10% nonimmune horse serum (NHS; Sigma-Aldrich) and 0.1% Triton X-100 (Sigma-Aldrich) for 2 h. Sections were then incubated for 18–24 h in primary antibody solution containing antisera detailed in Table 1. Each section was immunolabelled for CTB with one of the following: transient receptor potential vanilloid 1 (TRPV1; nociceptive afferents), calcitonin gene-related peptide (CGRP; expressed by many nociceptive and some myelinated afferents), or neurofilament 200 kDa (NF200; a marker of myelinated afferents). Sections were distributed in a 1:6 series to ensure comparable sampling for each antibody combination. Following a wash in 0.1 m PBS, sections were incubated for 2 h in combinations of appropriate species-specific secondary antibodies (Table 1) diluted in hypertonic PBS. Sections were cover-slipped using carbonate-buffered glycerol (pH 8.6) before viewing (Zeiss AxioImager M2, and Zeiss LSM 880 Fast Airyscan Confocal; Zeiss). Nucleated profiles of CTB+ somata were classified as positive or negative for each neural marker by directly viewing each section down the wide-field fluorescence microscope. Representative images were obtained using confocal microscopy for each immunohistochemical class of CTB+ somata.

**Table 1:** Primary and secondary antibodies

Primary antibodies						
RRID	Antigen		Host	Dilution	Supplier	Catalog number
AB_1658411	CGRP		Mouse	1:500	Abcam	AB81887
AB_11214092	ChAT		Goat	1:500	Millipore	AB144P
AB_258833	CTB		Rabbit	1:30,000 (sections); 1:3000 (iDisco clearing)	Sigma-Aldrich	C3062
AB_477257	NF200		Mouse	1:4000	Sigma-Aldrich	NO142
AB_90755	TH		Sheep	1:1000	Millipore	AB1542
AB_1624144	TRPV1		Goat	1:1000	Neuromics	GT15129
Secondary antibodies						
RRID	Antigen	Tag	Host	Dilution	Supplier	Catalog number

AB_2307351	Goat IgG	Cy3	Donkey	1:2000	Jackson ImmunoResearch	705-165-147
AB_2340433	Goat IgG	AF594	Donkey	1:500	Jackson ImmunoResearch	705-585-147
AB_2340846	Mouse IgG	AF488	Donkey	1:2000	Jackson ImmunoResearch	711-545-150
AB_2535789	Mouse IgG	AF594	Donkey	1:1000	Molecular Probes	A-21203
AB_2313584	Rabbit IgG	AF488	Donkey	1:1000	Jackson ImmunoResearch	711-545-152
AB_2307443	Rabbit IgG	Cy3	Donkey	1:3000	Jackson ImmunoResearch	711-165-152
AB_2536183	Rabbit IgG	AF647	Donkey	1:1000	Molecular Probes	A-31573
AB_10374882	Sheep IgG	AF647	Donkey	1:500	Molecular Probes	A-21448

CGRP, calcitonin gene-related peptide; ChAT, choline acetyltransferase; CTB, cholera toxin B subunit; NF200, neurofilament 200 kDa; TH, tyrosine hydroxylase; TRPV1, transient receptor potential vanilloid 1.

### Visualization of LUT afferents in sections of lumbosacral spinal cord

LUT afferents innervating the bladder body or proximal urethra were mapped in detail from three rats of each sex for each LUT region. Using the ventral roots to define segment boundaries, the L5-S2 spinal cord was subdissected into individual spinal segments, cryoprotected overnight in 0.1 m PBS containing 30% sucrose and then embedded in OCT compound. Transverse cryosections (40 μm) were collected in order, and alternate, free-floating sections immunolabelled for CTB with one or more other neural markers. Sections were washed in 0.1 m PBS, incubated in 0.1 m PBS containing 10% NHS and 0.5% Triton X-100 for 2 h, washed in 0.1 m PBS then incubated for 48 h at room temperature in primary antibody solution containing 0.1 m PBS, 2% NHS, 0.5% Triton X-100 and 0.1% sodium azide. Primary antibodies are described in Table 1 and comprised antibodies against CTB, choline acetyltransferase (ChAT), and tyrosine hydroxylase (TH). Following three further washes in 0.1 m PBS, sections were incubated for 4 h in combinations of appropriate species-specific secondary antibodies (Table 1) diluted in 0.1 m PBS containing 2% NHS, 0.5% Triton X-100, and 0.1% sodium azide. To enable the identification of spinal cord subregions, some sets of alternate sections were incubated with Neurotrace 435/455 (fluorescent Nissl stain; Invitrogen) concurrently with the secondary antibody. Sections were mounted on glass slides in rostrocaudal anatomic order and cover-slipped using carbonate-buffered glycerol (pH 8.6).

Spinal cord sections were imaged in their entirety (10× objective, tile scanned at 12 Bit, pixel scaling 0.645 × 0.645 μm, five z-steps at 5 μm) using a Zeiss AxioImager Z1 (Carl Zeiss Microscopy). Representative sections were also imaged using confocal microscopy (Zeiss LSM 880 Fast Airyscan). To facilitate comparison of homologous regions across images, all images showing only one side of the spinal cord have been oriented with the medial aspect on the left and lateral on the right of the image.

### Digital reconstruction of LUT afferents from alternate sections of spinal cord

To visualize the rostrocaudal distribution of sensory inputs from the LUT throughout the lumbosacral spinal cord, a maximum intensity orthogonal projection was first obtained for each channel of each section (ZEN 3.1 Blue Edition, Zeiss). Although the rostrocaudal order of the sections could be determined manually by inspecting their progressive change in overall size and gray matter shape, to increase efficiency we used a custom interactive script in MATLAB to order the sections according to their size. Final user input via an interactive gallery view of all ordered sections could check and, if necessary, re-order individual sections. Ordered alternate spinal cord sections were then aligned using TissueMaker (version 2021.1.1, MBF Bioscience), with distance between sections set at 80 μm (comprising 40-μm section thickness and 40 μm between sections). A small number of sections (less than five) were removed from the reconstruction for six spinal cords, because of damage or folding that impaired the alignment process (e.g., if cytoarchitectural and chemoarchitectural fiduciary markers were no longer visible). In cases where the section position could still be determined, the extra distance was inserted using the “missing section” option in TissueMaker. For the rare cases where the section position was indeterminable, extra distance was not inserted. Specific regions of gray matter were identified from (Watson et al., 2009).

### Quantification of LUT afferents in regions of interest (ROIs) of reconstructed spinal cord

Neurolucida 360 (version 2021.1.1, MBF Bioscience) was used to analyze neuronal processes labeled by CTB in the digitally reconstructed spinal cord datasets exported from TissueMaker. CTB granules appeared punctate throughout the afferent projections into the spinal cord. The *Detect Cells* function in Neurolucida 360 detects round objects within defined limits (minimum 1 μm, maximum 4 μm), based on their signal intensity compared with background. Using this function, CTB+ LUT afferents were segmented throughout the reconstructed spinal cord. This enabled visualization of axon patterning and provided a surrogate measure of afferent volume in selected ROIs (see below second paragraph of ‘Quantification of LUT afferents in regions of interest (ROIs) of reconstructed spinal cord’). CTB within neural processes appeared punctate, such that individual axons could usually not be resolved or traced.

Quantitation of CTB was performed throughout the length of the L6 and S1 spinal cord segments. The sacral dorsal commissural nucleus (SDCom) and sacral preganglionic nucleus (SPN) were selected for quantitative analysis, as both regions are major targets for LUT afferents (de Groat et al., 1981; Nadelhaft and Booth, 1984; Wang et al., 1998; Fowler et al., 2008). The SPN ROI was determined by the location of ChAT+ neurons, with the SPN boundary in each section drawn using the freeform contour tool in Neurolucida 360. The SDCom was defined using a rectangular contour, with the upper and lower boundaries determined by the dorsal limit of the central canal and the ventral edge of the dorsal corticospinal tract. The width of the SDCom contour aligned with the medial edges of the dorsal horn. This contour was applied to maintain consistency between sections where the chemoarchitectural boundaries of the SDCom were not readily apparent. This was because of either the absence of Nissl staining (only applied to some spinal cord sets) or spinal cord segments in which the SDCom has not previously been formally defined, such as L5 (Watson et al., 2009).

Using Neurolucida Explorer, the number of CTB puncta in each ROI and each section was determined throughout L6 and S1 segments. Retrograde tracing experiments cannot label precisely the same number of neurons in each animal, so we used the following strategy to aggregate data for each experimental group. To document the projection patterns of CTB-labeled neural processes along the L6-S1 cord, in each animal we first calculated the total number of CTB puncta for a specific ROI within the entire L6-S1 cord by summing the puncta counts for each section. For each ROI, in each section the CTB puncta were then expressed as a proportion of the total CTB for this ROI in this animal. Data were plotted as distance (μm) from the L6-S1 junction.

### Experiment design

Four experiments were performed, comprising neural tracing from bladder or urethra, in groups of female or male rats. Within each subject, measurements were made from ROIs (SPN or SDCom) in an ordered series of sections through spinal cords segments L6 and S1. Sacral preganglionic neurons do not distribute uniformly in a rostrocaudal column but are clumped and appear “ladder like” when viewed in horizontal sections. As this was consistent with large section-to-section variance, for each within-subject profile, data were aggregated using the area under the curve (AUC) of the profile within the L6 and S1 segments. Planned two group within-subject comparisons of L6 and S1 segments were made using a paired, two-tailed *t* test within each experiment (to avoid pooling variance of within-subject factors across experiments and potentially violating assumption of sphericity). Plots of the mean differences between L6 and S1 with 95% confidence intervals were used to visualize effect size as well as the variation of the paired comparisons.

Graphs were created in JMP 15.0 (SAS Institute) and data presented as the mean ± SEM. Statistical analyses were performed using IBM SPSS Statistics 27.0 (IBM).

### Visualization of LUT afferents in intact spinal cord

Spinal cord (L5-S2) was dissected from two anesthetized, intracardially perfused rats as described above (in ‘Tissue Collection’). Small lengths of ventral rootlets were retained with the spinal cord to identify segmental boundaries identified by origin of rootlets transitioning from L6 to S1 ventral roots. The large-volume clearing and immunolabelling method initially developed by Renier and colleagues formed the basis of our current clearing protocol (Renier et al., 2014). Samples were incubated in 1× Dulbecco’s phosphate-buffered saline (DPBS; Sigma-Aldrich; 6 × 15 min), dehydrated through a series of increasing concentrations of methanol in DPBS (50%, 80%, and 100%; 1.5 h each), bleached overnight (6% hydrogen peroxide in methanol, 4°C), then rehydrated through decreasing concentrations of methanol in DPBS (100%, 100%, 80%, and 50%; 1.5 h each). After washing in DPBS (1.5 h), samples were incubated for 36 h at room temperature in a blocking solution (DPBSG-T: DPBS with 0.2% gelatin, 0.5% Triton X-100, and 0.01% thimerosal). Samples were transferred to primary antibodies diluted with DPBSG-T containing 0.1% saponin, for 10 d at 37°C, washed (DPBS with 0.5% Triton X-100; DPBS-T; 6 × 15 min), then incubated in secondary antibody solution in DPBSG-T with 0.1% saponin for 4 d at 37°C. Primary antibodies used were ChAT with CTB, in combination with the appropriate secondary antibodies tagged with Cy3 and AF647, respectively (Table 1). Samples were washed in DPBS-T (6 × 15 min), dehydrated in methanol in DPBS (20%, 40%, 60%, 80%, 2 × 100%; 1 h each), then incubated overnight in 66% dichloromethane and 33% methanol at room temperature. The next day, spinal cords were incubated twice in 100% dichloromethane for 30 min, cleared with dibenzyl ether, then stored in fresh dibenzyl ether.

At least 3 h before microscopy, samples were immersed in ethyl cinnamate, as required for safe handling in the microscopy facility (Renier et al., 2016; Prahst et al., 2020). Samples were visualized in ethyl cinnamate with a light sheet fluorescence microscope (Ultramicroscope II, LaVision Biotec), using a 12× fixed zoom lens (MVX-10 Zoom Body, Olympus). Single-sided 3-sheet illumination was used with no dynamic focusing. Lasers 561 and 639 were used in conjunction with emission filters 620/60 and 680/30. The numerical aperture was 0.156. Image stacks were generated at 2-μm z-steps with 200-ms exposure per step. All mosaic acquisitions were performed with 10% overlap. Mosaic scans were stitched using the BigStitcher plugin to Fiji (Hörl et al., 2019). The resulting image stacks were then converted to Imaris file types using the Imaris File Converter (Bitplane) before being visualized in Imaris (Bitplane).

### Antibody characterization

The primary antibodies were acquired from the following sources (note that any information regarding specificity is obtained from the manufacturer):

1. CGRP (Abcam; AB81887; batch GR3283855-3; RRID: AB_1658411): immunoaffinity purified monoclonal antibody generated in mouse against rat-α CGRP.
2. ChAT (Millipore; AB144P; batch 2971003; RRID: AB_11214092): immunoaffinity purified polyclonal antibody raised in goat against the human placental enzyme; 70-kDa bands were detected in NIH/3T3 lysate Western blottings, matching the weight of ChAT protein.
3. CTB (Sigma-Aldrich; C3062; batch 048M4780V; RRID: AB_258833): polyclonal antibody raised in rabbit against *Vibrio cholerae*. The antiserum reacts versus cholera toxin, but not against staphylococcal enterotoxin A, staphylococcal enterotoxin B, and pseudomonas exotoxin A.
4. NF200 (Sigma-Aldrich; N0142; batch 17154802; RRID: AB_477257): immunoaffinity purified monoclonal antibody raised in mouse against C-terminal segment of enzymatically dephosphorylated pig NF200.
5. TH (Millipore; AB1542; batch 3192417; RRID: AB_90755): immunoaffinity purified polyclonal antibody raised in sheep against rat TH. Routine Western blot analysis detects a band of ∼62 kDa.
6. TRPV1 (Neuromics; GT15129; batch 402172; RRID: AB_1624144): immunoaffinity purified polyclonal antibody generated in goat against KLH-coupled synthetic peptide corresponding to amino acid residues 4–21 of vanilloid receptor 1 (TRPV1). Western blot analysis detects a bond of ∼115 kDa.

### Figure production

For representative images obtained with conventional wide-field fluorescence or confocal microscopy, small changes in levels were made in some panels to best represent the labeling as seen under the microscope. These adjustments were made in Photoshop (Adobe Creative Suite). For light sheet microscopy, structures were visualized with γ set to 1.4 in Imaris. Figures were constructed in Indesign (Adobe Creative Suite).

### Data sharing

Data supporting the findings of this study will be assigned a DOI and published under an open access license on https://sparc.science/. Movies illustrating the complete 3D datasets will also be available at this site.

## Results

### CTB labels major classes of LUT afferents

**Figure 1.**
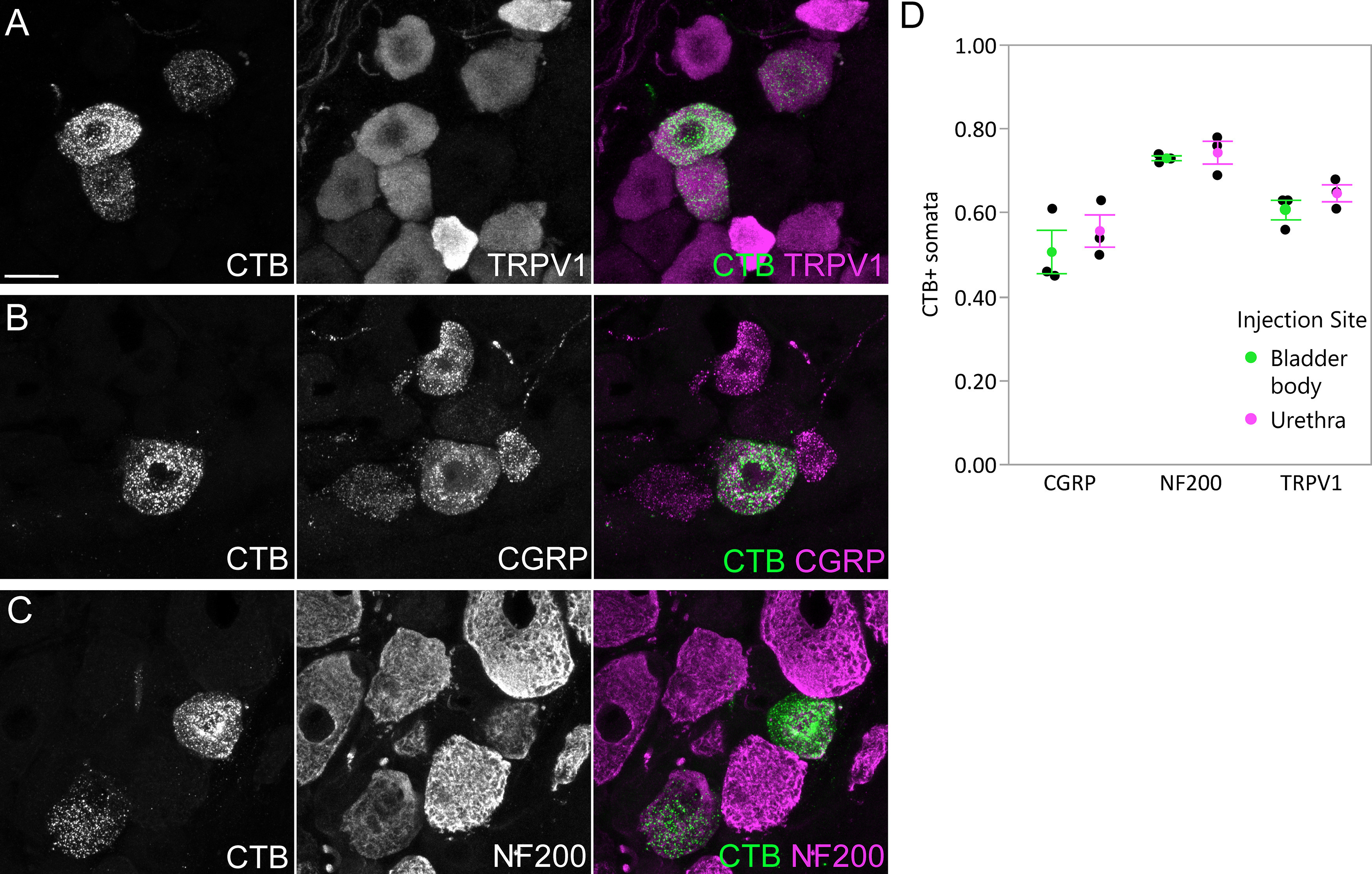
Uptake of CTB by afferents following intramural injection into the bladder body or proximal urethra. Confocal micrographs of L6 DRG cryosections immunolabelled for CTB and (***A***) TRPV1, (***B***) CGRP, or (***C***) NF200, following injection of the bladder body or urethra of male rats. ***D***, Proportion (mean ± SE) of CTB-labeled somata immunoreactive for TRPV1, CGRP, or NF200, following CTB injection into the bladder body or urethra. CGRP, calcitonin gene-related peptide; CTB, cholera toxin B subunit; NF200, neurofilament 200 kDa; TRPV1, transient receptor potential vanilloid 1. Scale bar: 20 μm.

Numerous somata in L6 DRG were labeled after CTB injection into the LUT of male rats (*n* = 3 bladder body, *n* = 3 urethra; 4-d transport time). Subpopulations of retrogradely labeled sensory neurons were immunolabelled for TRPV1, CGRP, or NF200 (Fig. 1*A–C*), as reported in previous studies using the fluorescent retrograde tracer, FluoroGold (Forrest et al., 2013). Results were similar for bladder and urethra afferents (Fig. 1*D*). Together, this confirmed that CTB was a suitable tracer for identifying both myelinated and unmyelinated LUT afferents.

### Afferent patterning varies widely between sections from the same animal, regardless of LUT injection site or sex

Pilot studies comparing CTB transport times of 4 and 7 d detected no difference in density or distribution of structures labeled in the spinal cord, so a transport time of 4 d was chosen for the primary studies described below. Following injections into either of the two LUT regions (bladder body, proximal urethra), bright, punctate CTB-labeled axons were visible in sections throughout the L6 and S1 cord and in both sexes. For brevity, from this point the CTB-labeled axons will be referred to as LUT afferents. Unless stated otherwise, all statements relating to the LUT afferents apply to both bladder and urethra afferents and to both sexes; as such, images illustrating these results are drawn from both regions and sexes, rather than demonstrating each feature for each LUT region and sex.

**Figure 2.**
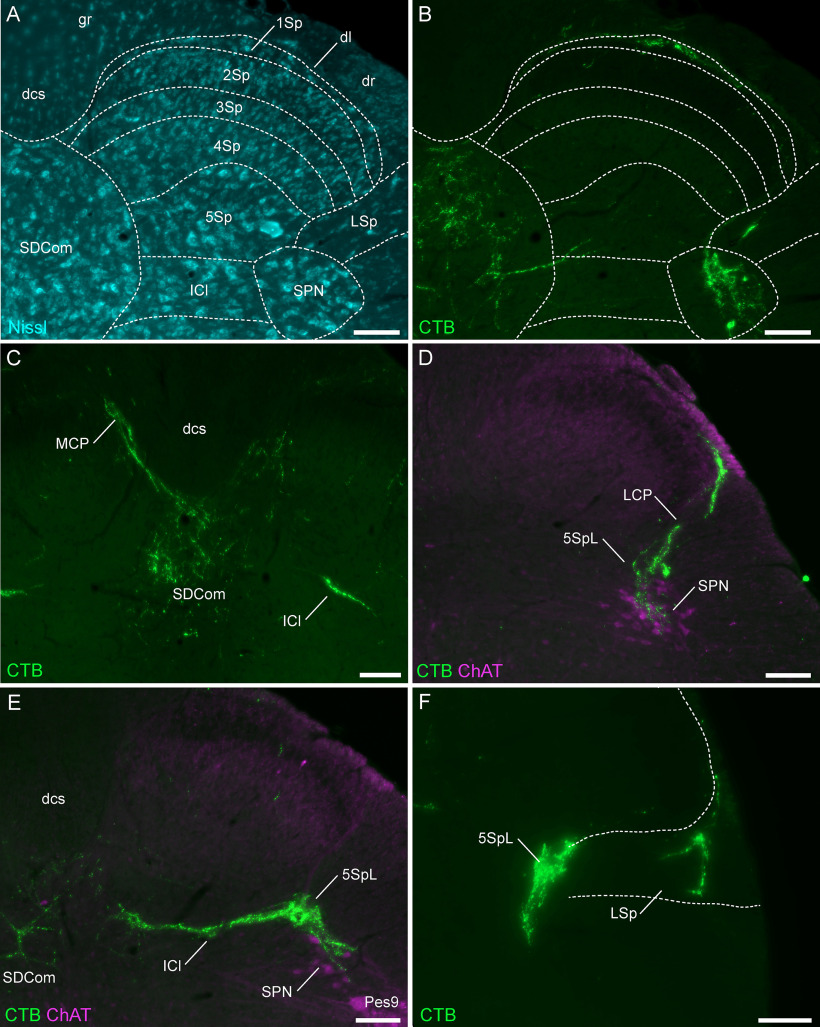
Major projections of CTB-labeled LUT afferents in the lumbosacral (L6-S1) spinal cord. ***A***, Cytoarchitecture shown by Nissl staining in an L6 spinal cord section and corresponding spinal cord regions from the atlas of Watson et al. (2009). ***B***, Urethra afferents (male) of the same section as ***A***, showing afferents in the SPN, SDCom, lateral spinal nucleus, and Lamina I of the dorsal horn. ***C***, Bladder afferents (male) in the medial collateral pathway, SDCom, and intercalated nucleus of the L6 spinal cord. ***D***, Urethra afferents (male) projecting in the lateral collateral pathway to lateral Lamina V of the dorsal horn and the SPN (co-labeled for ChAT) in the S1 spinal cord. ***E***, Bladder afferents (female) in the SPN, SDCom, and intercalated nucleus of the L6 spinal cord. ***F***, Bladder afferents (female) innervating lateral Lamina V of the dorsal horn and the lateral spinal nucleus in the L6 spinal cord. CC, central canal; ChAT, choline acetyltransferase; CTB, cholera toxin B subunit; dl, dorsolateral fasciculus; dcs, dorsal corticospinal tract; g, gracile fasciculus; ICl, intercalated nucleus; LCP, lateral collateral pathway; LSp, lateral spinal nucleus; LUT, lower urinary tract; MCP, medial collateral pathway; 1–10Sp, Laminae I–X of the spinal gray (dorsal horn); SDCom, sacral dorsal commissural nucleus; SPN, sacral preganglionic nucleus. Each image is oriented with dorsal at the top of the micrograph; the lateral dorsal horn is on the right for all panels except ***C***, midline view. Scale bars: 100 μm.

LUT afferents were mapped to previously defined subregions of L6-S1 spinal cord (Watson et al., 2009) illustrated in Figure 2*A*. The distribution of LUT afferents demonstrated the primary features of these afferents previously reported within the L6-S1 region (Jancsó and Maggi, 1987; Vizzard et al., 1995; Nadelhaft and Vera, 1996; Wang et al., 1998), i.e., tracts entering the spinal cord in the dorsolateral fasciculus, their trajectories at the lateral and medial boundaries of the dorsal horn (lateral and medial collateral pathways), and their respective regions of termination within the SPN and SDCom, as well as in the adjacent, lateral region of Lamina V and intercalated nucleus (Fig. 2*B–F*). Few LUT afferents were identified within the superficial dorsal horn (Laminae I–III; Fig. 2*B*,*D*). Occasionally, LUT afferents were observed to branch from the lateral collateral pathway to innervate the lateral spinal nucleus that lies immediately ventrolateral to the dorsal horn within the white matter (Fig. 2*F*).

Within spinal cord sections from each individual animal, there was a high degree of variability in the density and location of LUT afferents within each of these regions. The examples shown in Figure 2 represent the highest density of LUT afferents found in each region. Many sections from the same animals showed much lower density or absent innervation in one or more regions. This level of variability precluded a more detailed comparison of afferent patterning across the spinal cord segments.

### CTB-labeled structures associated with motor pathways

Within the lumbosacral spinal cord, there are two major types of motor pathways relevant to LUT function: parasympathetic preganglionic neurons that innervate pelvic ganglion neurons (e.g., to stimulate bladder contraction) and primary somatic motoneurons (e.g., that innervate sphincters and pelvic floor muscles). We identified these groups by their immunoreactivity for ChAT and their location (preganglionic neurons in the intermediolateral column and motoneurons in the ventral horn). The crural flexors, crural extensors, hamstring (Hm9) and gluteal (Gl9) motoneuron columns, which comprised the large lateral pool of motoneurons of Lamina IX in L5, diverged at the boundary of L6. In the ventral horn of L6, the Gl9 and Hm9 columns were located in the ventrolateral region and the pes motoneurons of Lamina IX (Pes9) in the dorsolateral region. The caudal edge of these motor columns aligned with the L6-S1 boundary. These structures are described further and illustrated below.

**Figure 3.**
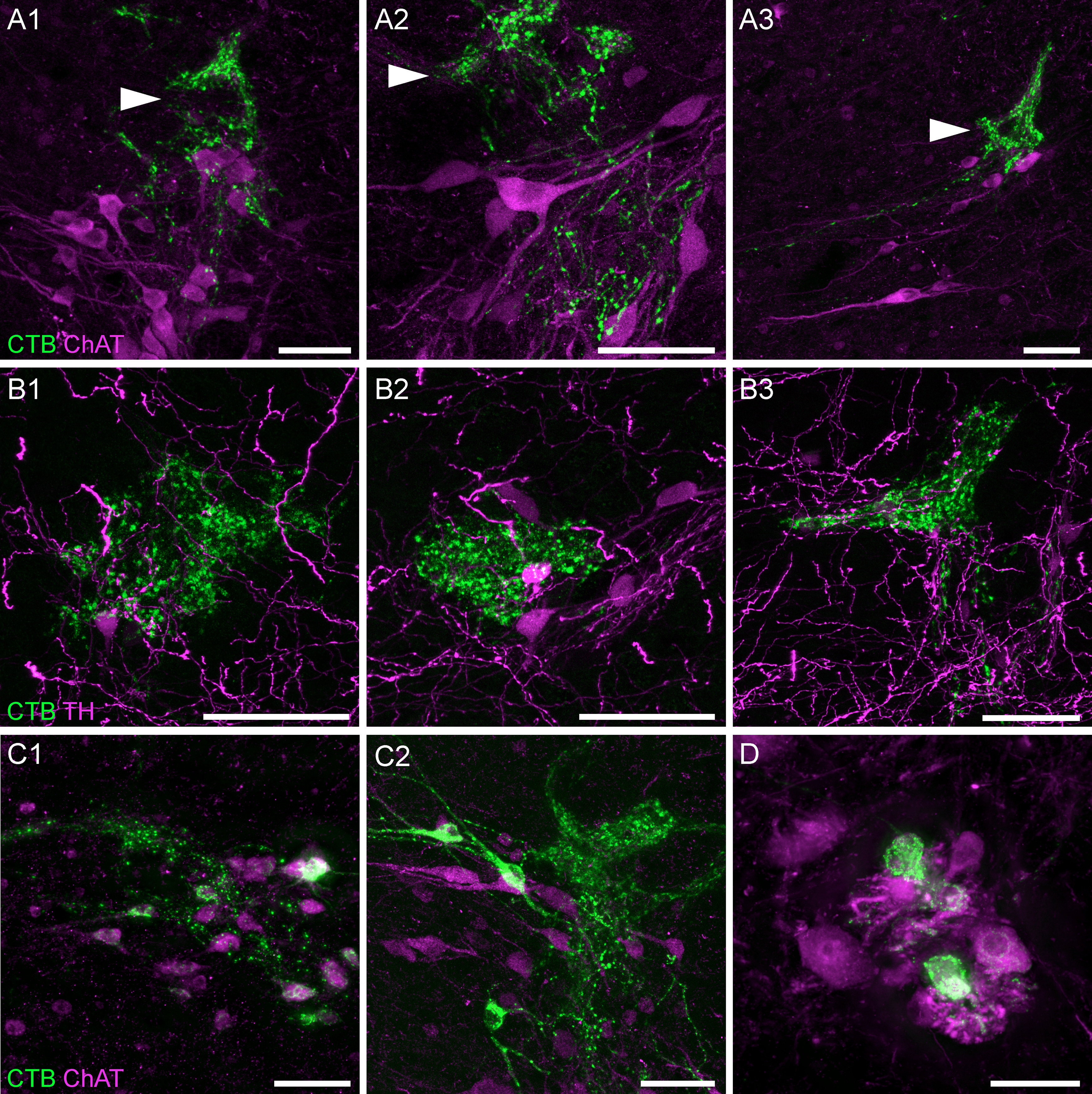
LUT afferent innervation of the SPN and surrounding regions. ***A***, Confocal micrographs demonstrate the close apposition of CTB-labeled bladder afferents to cholinergic (ChAT) neurons in the SPN of a male rat; most of the afferent innervation in this region is located immediately dorsal to the SPN, in lateral Lamina V (arrowheads). ***B***, Confocal micrographs of bladder afferent innervation of TH-positive neurons in lateral Lamina V of a male rat. ***C***, CTB-labeled cholinergic neurons of the SPN in (***C1***) male and (***C2***) female rats following urethra injections of CTB. ***D***, CTB-labeled neurons in the dorsolateral motor nucleus of a female rat after CTB injection into the urethra. ChAT, choline acetyltransferase; CTB, cholera toxin B subunit; LUT, lower urinary tract; TH, tyrosine hydroxylase. Scale bars: 50 μm.

LUT afferents were most dense near and within the SPN (Figs. 2*B*,*D*,*E*, 3). Within this nucleus, confocal microscopy demonstrated close appositions between LUT afferents and the somata and dendrites of preganglionic neurons (Fig. 3*A*). A higher density of LUT afferents was present within lateral Lamina V, immediately dorsal to the SPN (Fig. 3*A*, arrowheads). This region has previously been identified as the location of inhibitory and excitatory interneurons involved in micturition (Vizzard et al., 1995; Araki and de Groat, 1996; de Groat et al., 2015), some of which express TH (Hou et al., 2016a, 2021; Wiedmann et al., 2021). Confocal microscopy confirmed close associations between LUT afferents and TH neurons in this region, however the majority of LUT afferents were not near TH neurons (Fig. 3*B*).

In some experiments, we also identified L6 and S1 preganglionic neuron somata labeled with CTB (Fig. 3*C*). Specifically, their prevalence varied with the LUT injection site, being rare following CTB injection into the bladder, but more common following injection of the urethra (especially in females). Because CTB does not cross synapses, the most likely explanation for this labeling is that some autonomic ganglia (the termination sites of preganglionic neurons) were present near the injection site. Most of the LUT-projecting autonomic ganglion neurons are located within the major pelvic ganglia that are distant from the CTB injection sites, but several microganglia are located near the surface of the bladder neck and proximal urethra (Arellano et al., 2019) and single or small groups of ganglion neurons are dispersed within the bladder wall (Forrest et al., 2014), these could potentially be exposed to CTB.

CTB-labeled motoneurons were identified in several female rats following injection of CTB into the urethra (Fig. 3*D*). These neurons were consistently located in segment L6, in the motoneuron pool of Lamina IX that innervates the urethral rhabdosphincter (Schrøder, 1980; Nadelhaft and Vera, 1996). This indicates that the urethral rhabdosphincter was exposed to a small amount of CTB. Except for rare neurons, labeling of neurons in this nucleus was not identified after urethra injection of CTB in male rats or after bladder injections.

### Preganglionic neurons are absent from the rostral half of spinal segment L6

**Figure 4.**
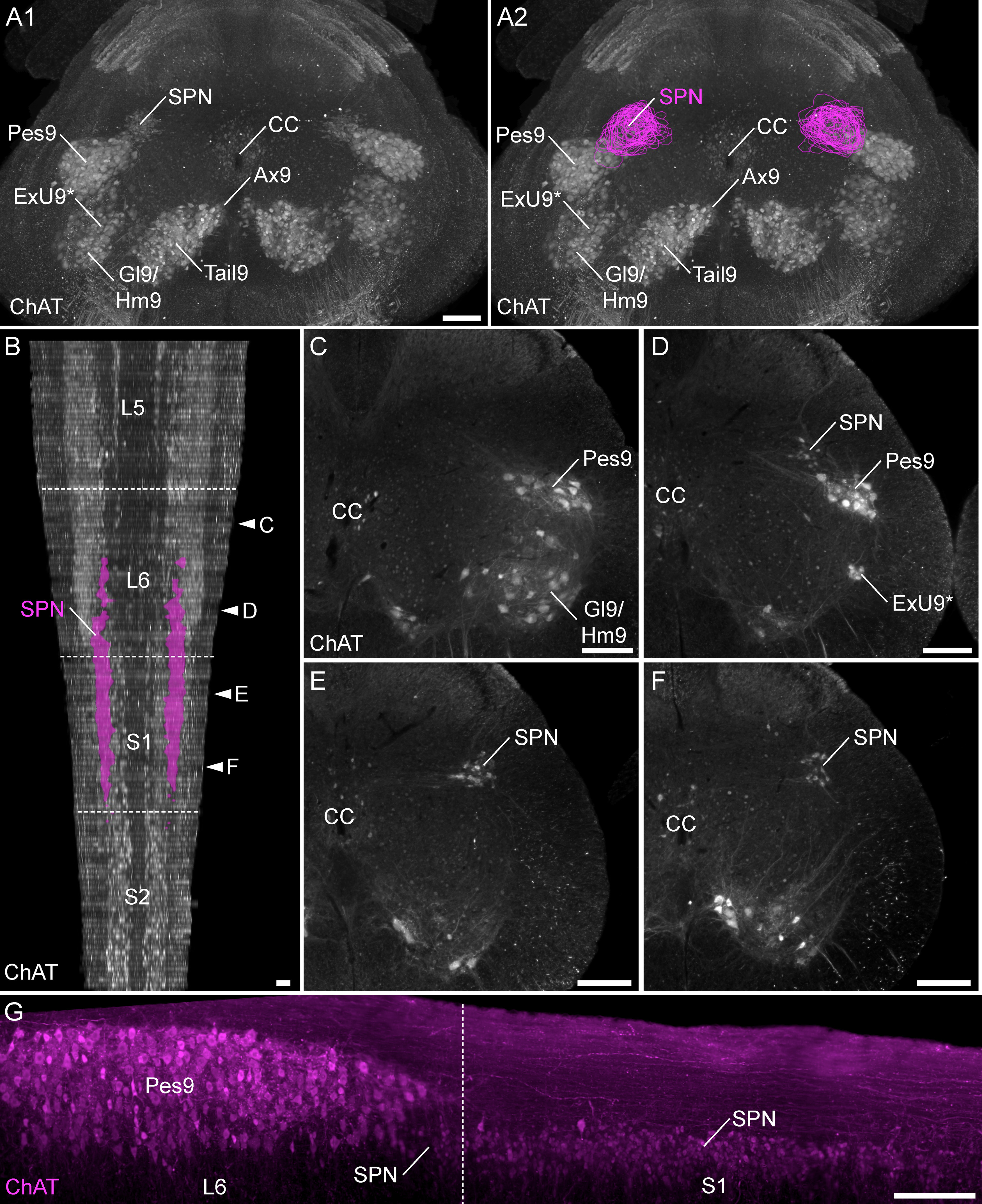
The SPN location within the lumbosacral spinal cord. ***A***, A 3D reconstruction was created from the digital alignment of a 1:2 series of 121 cryosections of the lumbosacral spinal cord (female rat). ***A1***, Transverse view of the aligned L6 and S1 segments showing motoneuron nuclei and preganglionic autonomic neurons identified via ChAT immunolabelling. Preganglionic neurons are less strongly immunolabelled than motoneurons. ***A2***, The boundary of the SPN in each section was identified at higher magnification and delineated manually on each section. ***B***, The full 3D reconstruction is shown here in the horizontal plane, demonstrating that the SPN is present in both L6 and S1 segments but does not extend for the full length of L6. Examples of individual sections from four locations along the cord are indicated by arrowheads in ***C–F***. ***C–F***, Individual cryosections demonstrating that preganglionic neurons are absent in rostral L6 (***C***) but present in (***D***) caudal L6, (***E***) rostral S1, and (***F***) caudal S1. Specific motoneuron nuclei confirm segment position; these nuclei reduce in cell number in the caudal direction until they end at the L6-S1 junction. The rostral boundary of the SPN in L6 coincides with the narrowing of the Pes9 nucleus, approximately halfway along the L6 segment, as also observed in (***G***) light sheet image stacks of iDISCO-cleared intact lumbosacral spinal cord (male rat). Motoneurons of Lamina IX: Ax9 (axial), ExU9* (external urethral sphincter), Gl9 (gluteal), Hm9 (hamstring), Pes9 (pes), Tail9 (tail); CC, central canal; ChAT, choline acetyltransferase; SPN sacral preganglionic nucleus. *, the location of ExU9 is indicated according to Schrøder (1980), McKenna and Nadelhaft (1986), Nadelhaft and Vera (1996, 2001), and Xu et al. (2007). Scale bars: 200 μm.

To identify specific patterning of LUT afferent projections within and between L6 and S1 segments, we digitally reconstructed this region of cord from alternate serial transverse sections (Fig. 4*A*,*B*). In many of these animals, the entire length of segments L5 and/or S2 were also included. This approach revealed distinct features within rostral and caudal regions of the L6 segment.

**Figure 5.**
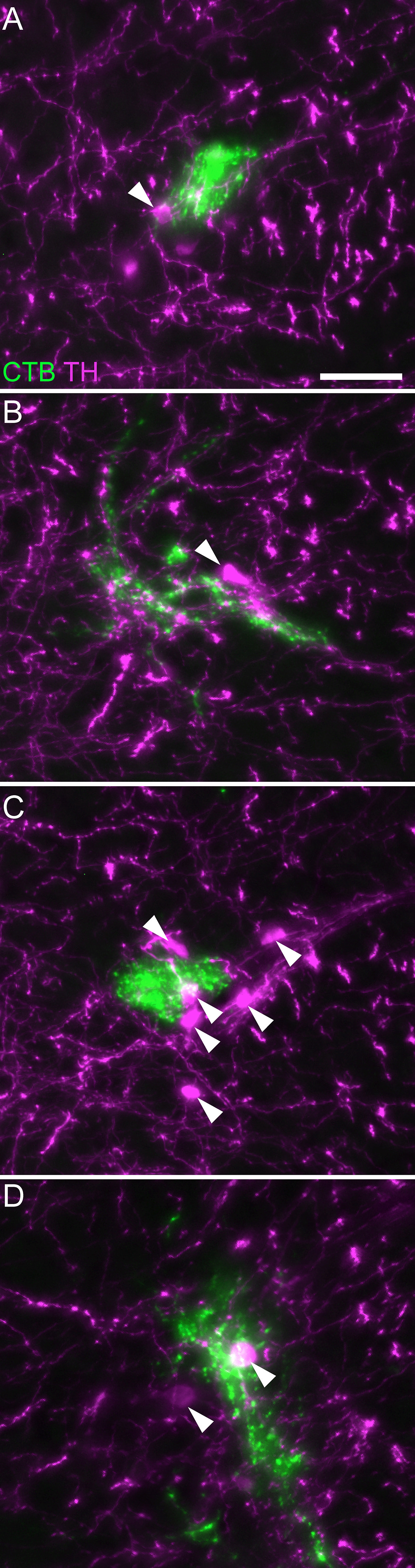
Association of LUT afferents with TH neurons in lateral Lamina V. CTB-labeled bladder afferents innervating lateral Lamina V in (***A***) rostral L6, (***B***) mid-L6, (***C***) caudal L6, and (***D***) rostral S1 of a female rat. Many but not all TH neurons show close associations with LUT afferents. Arrowheads indicate examples of TH-positive neurons. CTB, cholera toxin subunit B; LUT, lower urinary tract; TH, tyrosine hydroxylase. Scale bar: 50 μm.

First, we found that the SPN does not extend through the entire length of L6 and S1 segments. Instead, the SPN commences only within the most caudal half of L6, at which point the nucleus of Pes9 begins to reduce in size (Fig. 4*B–D*). From this point, the SPN extends continuously to the caudal end of segment S1 (Fig. 4*E*,*F*). In an iDISCO-cleared lumbosacral spinal cord, the overlap of the SPN and Pes9 confirmed the relative distribution of the SPN and the Pes9 motor column (Fig. 4*G*). Second, the population of TH neurons in lateral Lamina V comprised a discontinuous column throughout L6 and S1 segments (Fig. 5). These neurons were still present in the rostral half of L6 (where the SPN was absent) but were sparser at this level (Fig. 5*A*). Together, these observations demonstrate the importance of defining the precise spinal level of afferent innervation, especially within the L6 segment.

### Afferents innervating the bladder and urethra extend beyond spinal levels containing preganglionic neurons

**Figure 6.**
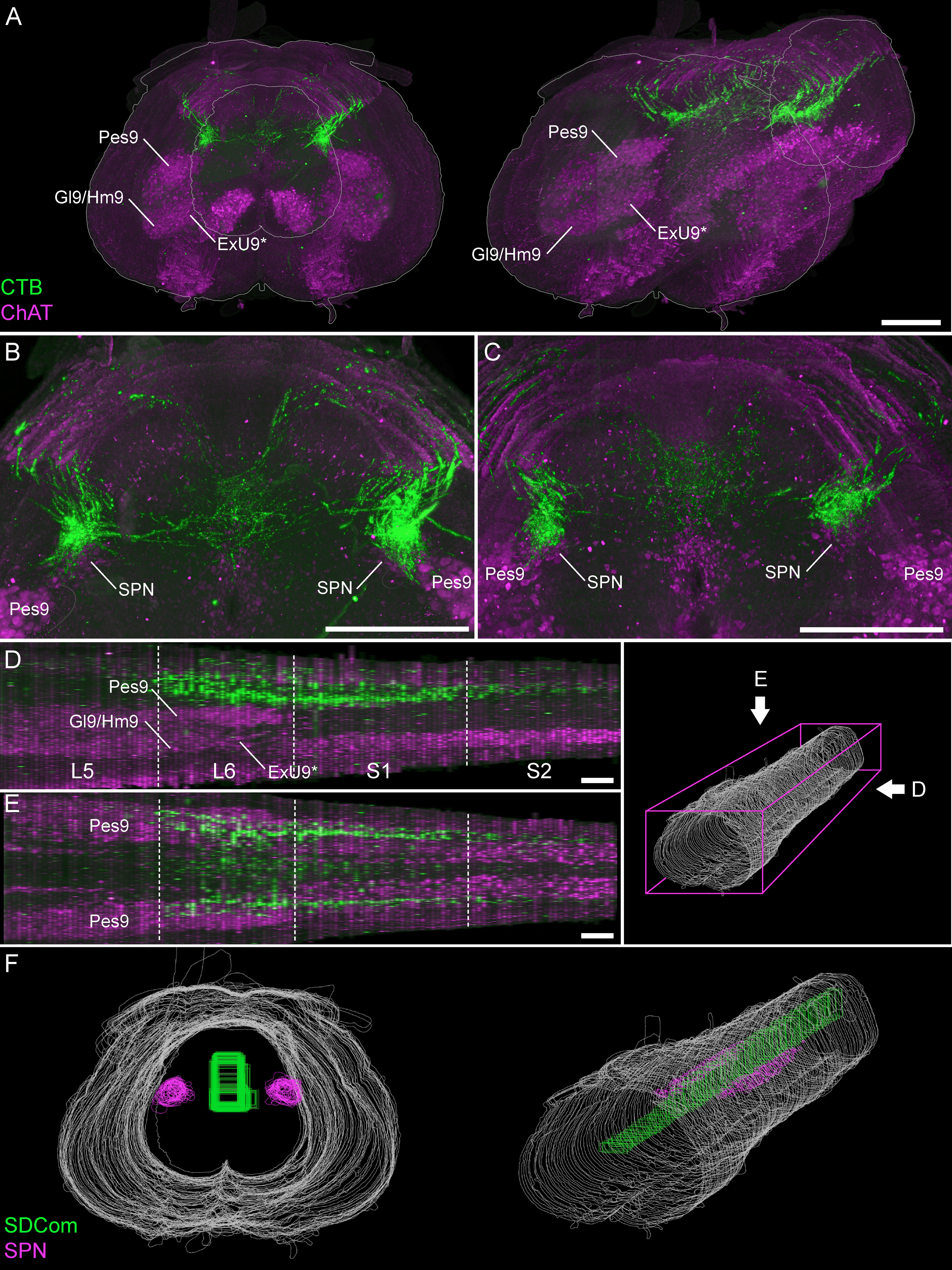
Visualizing the rostrocaudal distribution of bladder afferents in 3D reconstructions of lumbosacral spinal cord. ***A***, Visualization of 100 ordered and aligned sections in a single view along the rostrocaudal axis of the spinal cord (female rat), oriented with the rostral boundary at the front of the field of view in transverse (***A1***) and oblique (***A2***) views. Motor and preganglionic autonomic neurons were visualized by ChAT. ***B***, ***C***, Bladder afferents in a female (***B***) and male (***C***) rat, shown in a maximum intensity projection of the 3D reconstructed lumbosacral spinal cord. Sagittal (***D***) and horizontal (***E***) views of bladder afferent innervation (male rat) each demonstrate the rostrocaudal limits of LUT afferent innervation, indicated by the segment boundaries and the location of the lumbosacral motor nuclei. Schematic on the right shows the viewing orientation of ***D***, ***E***. ***F***, For further visualization and quantitative studies, the perimeters of individual sections were outlined (white) and specific ROIs defined, such as the SPN (magenta) and the SDCom (green). Motoneurons of Lamina IX: ExU9* (external urethral sphincter), Gl9 (gluteal), Hm9 (hamstring), Pes9 (pes). *, the location of ExU9 indicated according to Schrøder (1980), McKenna and Nadelhaft (1986), Nadelhaft and Vera (1996, 2001), and Xu et al. (2007). ChAT, choline acetyltransferase; CTB, cholera toxin B subunit; LUT, lower urinary tract; SDCom, sacral dorsal commissural nucleus; SPN, sacral preganglionic nucleus. Scale bars: 500 μm.

Our 3D reconstructions not only revealed distinct features within rostral and caudal regions of the L6 segment (see above, in ‘Preganglionic neurons are absent from the rostral half of spinal segment L6’) but also facilitated a more detailed analysis of LUT afferent projections (Fig. 6). Examples of this afferent patterning are demonstrated in various orientations of the reconstructed cord in Figure 6*A–E*, and the ROIs used for our quantitative studies in Figure 6*F*. Most primary afferents innervating the LUT originate from L6-S1 DRG (Keast and de Groat, 1992; Nadelhaft and Vera, 1996), therefore in this study we anticipated a high concentration of afferent terminations within both spinal segments. However, from our initial results summarized above (in ‘Afferent patterning varies widely between sections from the same animal, regardless of LUT injection site or sex’), we predicted that afferents would be absent from rostral L6 that was devoid of autonomic preganglionic neurons. Instead, we found that LUT afferents were not only present in rostral L6 but also projected to spinal segments with no preganglionic neurons, i.e., the adjacent segments of caudal L5 and rostral S2 (Fig. 6*D*,*E*). Therefore, our more detailed descriptions below of the primary features of LUT afferents extend across segments L5-S2 and have distinguished the rostral and caudal halves of L6.

Many of the qualitative features are similar across both LUT regions and sexes, which have been summarized together below. Unless stated otherwise, all features were common to both bladder and urethra afferents and to male and female rats.

From their point of entry to the dorsal horn, the LUT afferents project in two primary routes: the lateral collateral pathway that innervates the SPN and adjacent lateral region of Lamina V, and the medial collateral pathway that innervates the SDCom. For bladder but not urethra afferents, there were also finer projections to the lateral spinal nucleus within the white matter adjacent to the dorsal horn; this projection was most prominent in caudal L6, sparse in rostral L6 and absent from L5, S1 and S2.

#### The lateral collateral pathway and its projections

**Figure 7.**
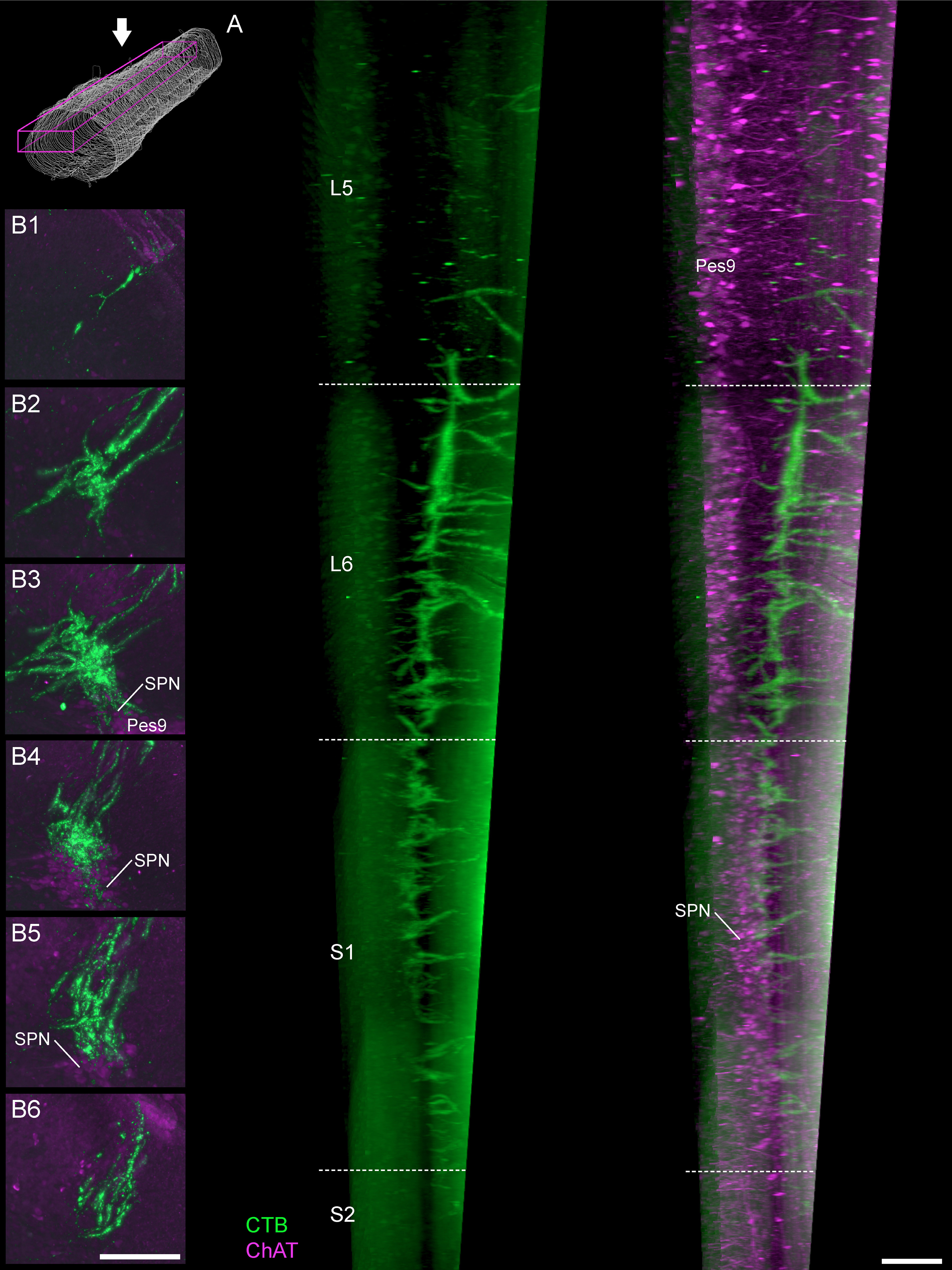
Projections of LUT afferents in the lateral collateral pathway. ***A***, In a parasagittal view of the cleared lumbosacral spinal cord, bladder afferents (male rat) show distinct gradations in density of the lateral collateral pathway projection. This extends from caudal L5 to rostral S2 but is most dense across the length of the L6 segment. Schematic shows viewing orientation. ***B***, Maximum intensity projections of discrete portions of the reconstructed spinal cord datasets reveal the distribution of bladder afferents (female rat) at different levels of the spinal cord: (***B1***) caudal L5, (***B2***) rostral L6, (***B3***) caudal L6, (***B4***) rostral S1, (***B5***) caudal S1, and (***B6***) rostral S2. This includes regions more proximal (***B1***, ***B2***) and more caudal (***B6***) to the SPN, as demonstrated by immunoreactivity for ChAT; ChAT, choline acetyltransferase; CTB, cholera toxin B subunit; LUT, lower urinary tract; Pes9, Pes motoneurons of lamina IX; SPN, sacral preganglionic nucleus. Scale bars: 200 μm.

LUT afferents are found in the lateral collateral pathway from the caudal region of L5 to the rostral region of S2 (Fig. 7). The densest projection of this pathway is found along the full extent of L6 (Fig. 7*A*). From this pathway, the LUT afferents expand into large terminal fields within lateral Lamina V and the SPN, where present; in L5 and S2 this forms smaller terminal fields, commonly restricted to small, tapered regions at the most dorsal boundary of Lamina V (Fig. 7*B*).

Of the two primary targets of the lateral collateral pathway within L6-S1, i.e., the SPN and lateral Lamina V, the highest density of LUT afferents occurred in lateral Lamina V. This occurred along the complete length of L6 and S1, even in rostral L6 where preganglionic neurons were absent (Fig. 7*B*). The SPN was also innervated along its full extent, i.e., extending from the caudal half of L6 and throughout S1 (Fig. 7*B*). Overall, LUT afferents were denser in the dorsal than the ventral SPN, although there was wide variation of SPN innervation between sections so this aspect would have been especially difficult to deduce without 3D reconstruction.

#### The medial collateral pathway and its projections

**Figure 8.**
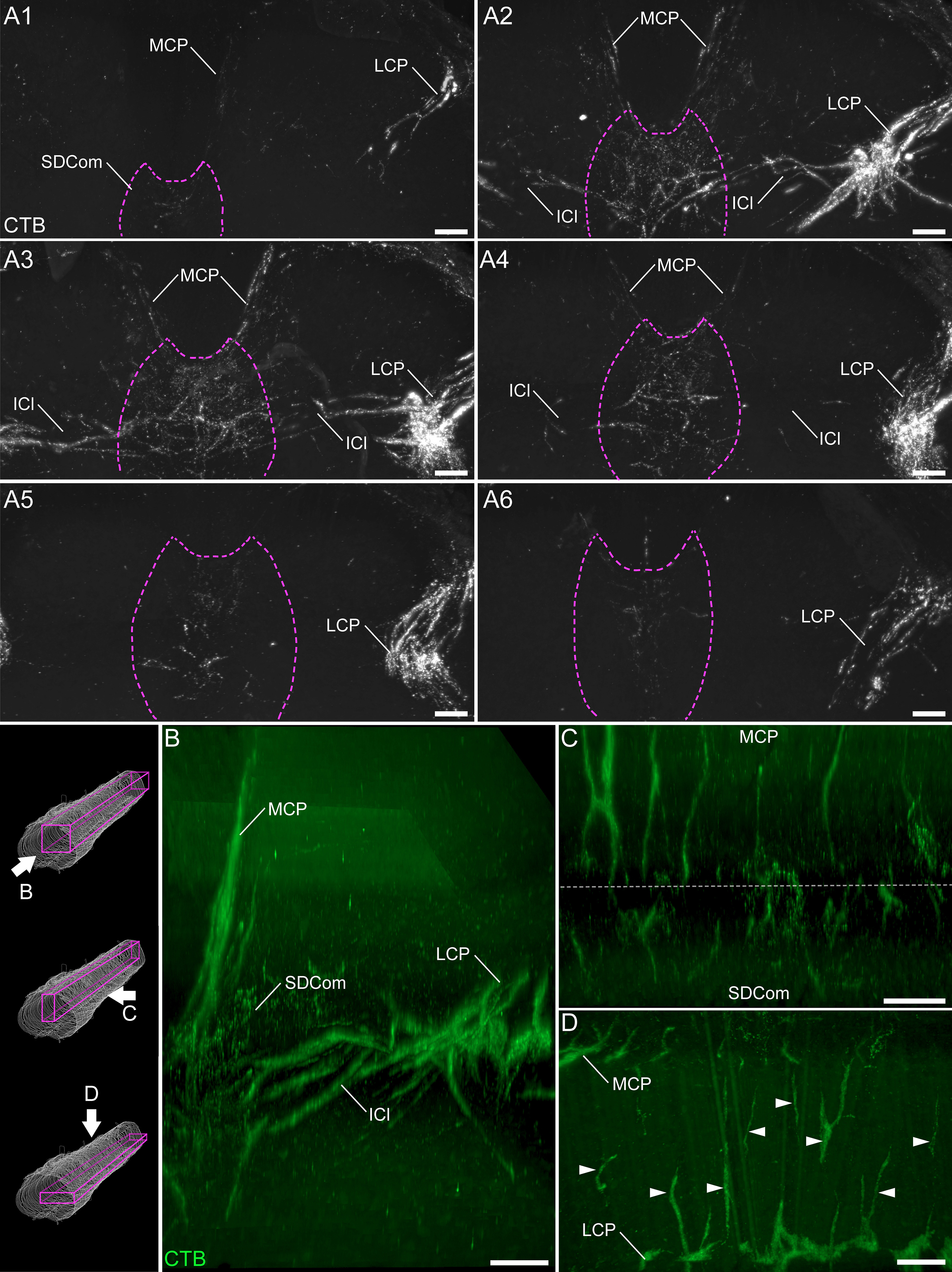
Projections of LUT afferents in the medial collateral pathway. ***A***, Discrete portions of the reconstructed spinal cord datasets were visualized with maximum intensity projections of bladder afferents (female rat), focusing on the medial collateral pathway, SDCom, and intercalated nucleus: (***A1***) caudal L5, (***A2***) rostral L6, (***A3***) caudal L6, (***A4***) rostral S1, (***A5***) caudal S1, and (***A6***) rostral S2. ***B***, In cleared lumbosacral spinal cord, individual tracts of the medial collateral pathway are most prominently observed in the parasagittal view. In the horizontal (***C***) and transverse (***D***) views, the LUT afferent innervation of the intercalated nucleus between the SDCom and the lateral collateral pathway are revealed. Schematic on ***B***, left, shows the viewing orientation of B-D. ICl, intercalated nucleus; LCP, lateral collateral pathway; MCP, medial collateral pathway; ChAT, choline acetyltransferase; CTB, cholera toxin B subunit; LUT, lower urinary tract; SDCom, sacral dorsal commissural nucleus. Scale bars: 100 μm.

The second primary trajectory of LUT afferents was the medial collateral pathway, which was generally a less prominent structure than the lateral collateral pathway. The medial collateral pathway was present across a similar length of spinal cord as the lateral collateral pathway, i.e., a consistent, strong density across L6 and S1, with much finer tracts in caudal L5 and in S2 (Fig. 8*A*). As previously reported (Wang et al., 1998), this pathway appeared to be the primary source of LUT afferents to the SDCom, demonstrated by the close correlation of LUT afferent density within the medial collateral pathway and the SDCom, across spinal segments (Fig. 8*A*). We were unable to confirm this projection by direct tracing of individual axons from the medial collateral pathway to the SDCom, because of the punctate nature of CTB distribution within axons.

In assessing the LUT afferent innervation of the SDCom, we noted that the size of this region varies between spinal segments, progressively increasing in size from rostral to caudal L6, then decreasing along the sacral segments. Moreover, LUT afferent innervation generally extended beyond the formal boundaries of the SDCom, into medial Laminae IV and V (see below). LUT afferents innervated SDCom throughout L6 and S1 cord but were denser in L6 than S1 (Fig. 8*A2–A5*). However, because SDCom is smaller in S1 than L6, a similar number of LUT afferents may target this region in each spinal segment. LUT afferents were present but less dense in the SDCom of L5 and S2 (Fig. 8*A1*,*A6*).

#### Additional gray matter subregions

LUT afferents were present medial to the SPN, including the intercalated nucleus that lies immediately dorsal to Lamina VII (Figs. 2*C*,*E*, 8*B–D*). This region contains long dendritic projections from the SPN that extend toward the SDCom (Nadelhaft and Booth, 1984). The punctate nature of LUT afferent labeling generally precluded direct tracing of individual axons through the gray matter, so it was not possible to confidently define the source (lateral or medial collateral pathways) of LUT afferents in the intercalated nucleus. Irrespective of their origin, the innervation of this region by LUT afferents varied between spinal segments, generally being most dense in caudal L6, less in rostral L6 and S1, and absent from L5 and S2 (Fig. 8*A*).

LUT afferents were also present in medial Lamina IV, adjacent to the SDCom. This region was innervated throughout L6 (with higher density in caudal than rostral L6), less in S1 and very sparse in S2. In L5, this region was not innervated by LUT afferents (Fig. 8*A*).

More superficial laminae of the dorsal horn (Laminae I–III) were consistently innervated by LUT afferents (Figs. 2*B*,*D*, 6*B*,*C*), but this was sparse compared with the gray matter regions described above (in ’The lateral collateral pathway and its projections’ and ‘The medial collateral pathway and its projections’). This sparse innervation occurred throughout L6 but was even less in S1 and barely detectable in L5 and S2. These segmental differences were apparent for both bladder and urethra afferents.

### Quantification of bladder and afferent innervation reveals distinct weightings of projections to different spinal segments and subregions

**Figure 9.**
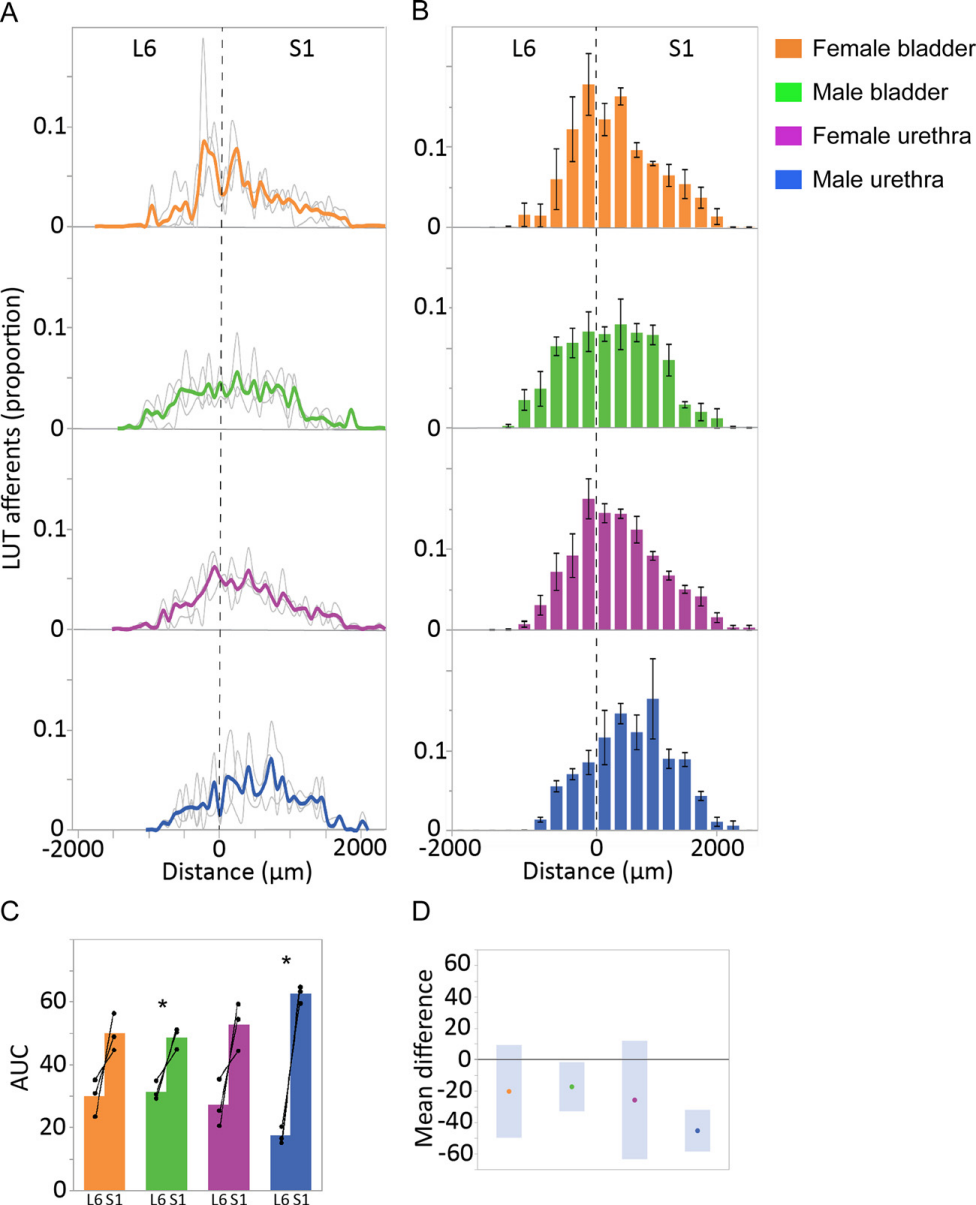
Quantitation of LUT afferent innervation of the SPN. ***A***, CTB-labeled afferents in each section, segmented and quantified as puncta, are expressed as the proportion of the total number of CTB puncta in the SPN of the same section. Data shown for individual animals (gray lines) and group mean (bold colors) and plotted as distance (μm) from the L6-S1 junction (0 μm, vertical broken line). ***B***, Data from individual sections was summed (three sections per bin) to better visualize overall distribution patterns (*n* = 3 rats per group, mean ± SE). ***C***, Total afferent innervation of the SPN in L6 and S1 segments determined by AUC, shown for individual animals and group data (mean ± SE) for each of the four experimental groups. In each animal, regardless of LUT region and sex, a larger number of afferent structures were found in S1 than L6. This segmental difference was also detected in analysis of the grouped data for the male bladder and male urethra groups [*p* = 0.042 (male bladder) and *p* = 0.005 (male urethra), paired two-tailed *t* test] but could not be detected for either of the female groups (female bladder, female urethra) where there was more intersubject variation in AUC measurements. ***D***, Plot of difference in group means (dots) and 95% confidence intervals (gray bars) for AUC measurements in SPN of L6 and S1 spinal cord. LUT, lower urinary tract; SPN, sacral preganglionic nucleus.

**Figure 10.**
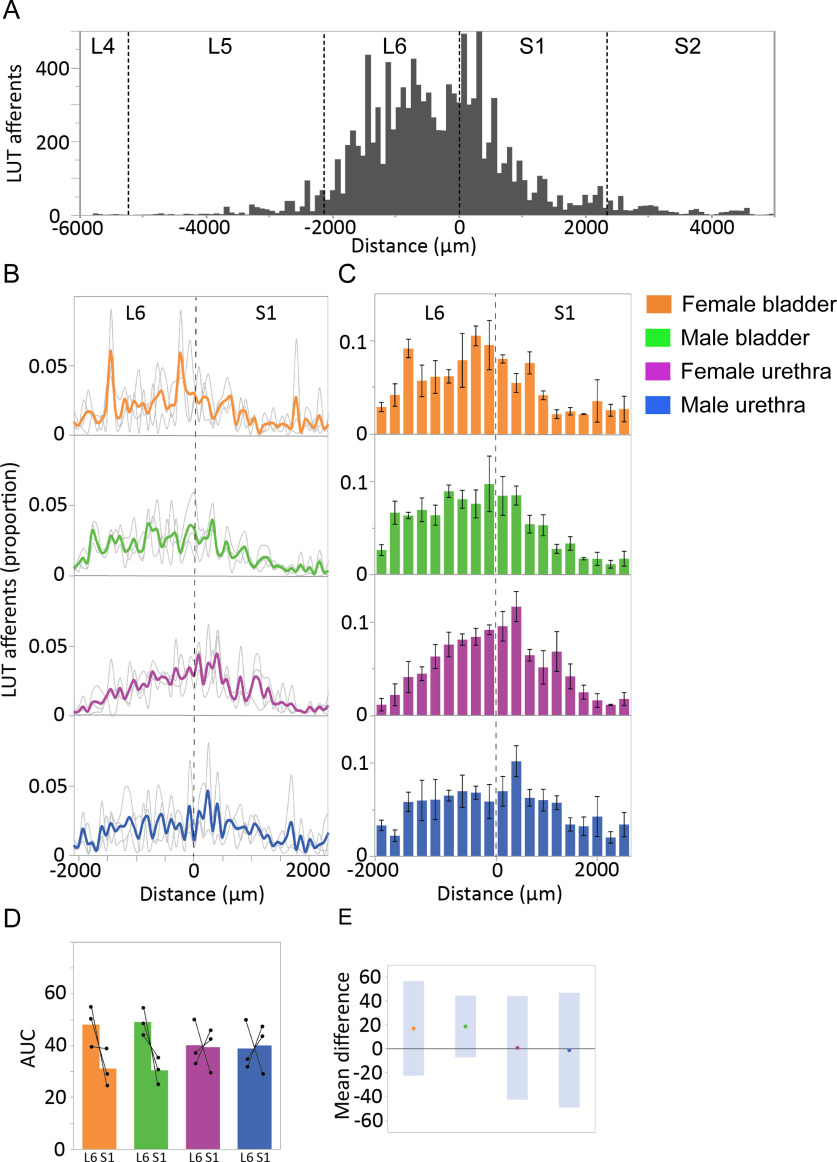
Quantitation of LUT afferent innervation of the SDCom. ***A***, The innervation of the SDCom extends beyond the L6 and S1 spinal cord segments, as exemplified in a dataset (bladder afferents, female rat) in which aligned cryosections were collected from caudal L4 until the caudal limit of S2. This also demonstrates the strong innervation of this nucleus in the rostral half of L6, a spinal level where preganglionic neurons are absent. ***B***, CTB-labeled afferents in each section, segmented and quantified as puncta, are expressed as the proportion of the total number of CTB puncta in the SDCom of the same section. Data shown for individual animals (gray lines) and group mean (bold colors) and plotted as distance (μm) from the L6-S1 junction (0 μm, vertical broken line). ***C***, Data from individual sections was summed (three sections per bin) to better visualize overall distribution patterns (*n* = 3 rats per group, mean ± SE). ***D***, Total afferent innervation of the SDCom in L6 and S1 segments determined by AUC, shown for individual animals and group data (mean ± SE) for each of the four experimental groups. No significant difference between L6 and S1 segments were detected in any of the four groups (paired, two-tailed *t* test). ***E***, Plot of difference in group means (dots) and 95% confidence intervals (gray bars) for AUC measurements in the SDCom of the L6 and S1 spinal cord. LUT, lower urinary tract; SDCom, sacral dorsal commissural nucleus.

LUT afferents in digitally reconstructed spinal cord (L6-S1) were segmented, then quantified in the SPN (Fig. 9) and SDCom (Fig. 10). The locations of these regions are illustrated in Figure 6*F*. For each of these regions, the total amount of afferent innervation (defined as number of punctate structures) was quantified in each section, then summed across the total number of L6 and S1 sections; the afferent labeling in each section was then expressed as a proportion of the total afferent labeling for that region, across the entire L6-S1 cord (Figs. 9*A*, 10*B*). This approach allowed us to visualize the afferent distribution across the length of each spinal segment and to compare data across animals, considering differences in the total amount of LUT afferent labeling because of limitations in tracer injection methodology. This data also illustrated the variations in afferent innervation between adjacent sections that was visible under the microscope, as described above (in ‘Afferents innervating the bladder and urethra extend beyond spinal levels containing preganglionic neurons’). To further improve our visualization of afferent patterning, we binned the data (data summed from three sections per bin; Figs. 9*B*, 10*C*) and compared innervation across each segment (AUC for summed data from individual sections; Figs. 9*C*, 10*D*).

Inspection of the individual animal and experiment binned data demonstrated the distribution of bladder and urethra afferents for both sexes across the SPN in both L6 and S1 segments (Fig. 9*A*,*B*). This indicated the maximum rostro-caudal density of innervation was around the L6-S1 boundary for bladder afferents in both sexes and urethra afferents in female rats. Further inspection of individual subject data from each segment (Fig. 9*C*) showed that in the SPN of each animal, regardless of LUT region and sex, a larger number of afferent structures were found in S1 than L6. This segmental difference was also apparent in the group data for the male bladder and male urethra experiments [L6 vs S1: *p* = 0.042 (male bladder) and 0.005 (male urethra); paired two-tailed *t* test]. This was further demonstrated by plotting the group mean differences using the mean and 95% confidence intervals (Fig. 9*D*). For each of the two female experiments (female bladder, female urethra) an L6-S1 segmental difference could not be detected, potentially because of the greater intersubject variation in AUC measurements (Fig. 9*C*,*D*).

Assessment of SDCom (Fig. 10) showed a different patterning of LUT afferent innervation. In datasets that contained sections from L5 and S2 (example shown in Fig. 10*A*), LUT afferent innervation could be seen to extend not only across the entire segments of L6 and S1, but also projected into L5 and S2 (although at a much lower density than in L6 and S1). As indicated by our earlier qualitative analysis (see above, in ‘Afferents innervating the bladder and urethra extend beyond spinal levels containing preganglionic neurons’), the individual subject (Fig. 10*B*) and group binned data (Fig. 10*C*) demonstrated that bladder and urethra afferents from each sex extended throughout the L6 and S1 regions that contained preganglionic neurons, in addition to more rostral L6 that did not contain the SPN. Afferent projections to the SDCom did not demonstrate the aggregation around the L6-S1 boundary we observed for afferents innervating the SPN. Further inspection of individual subject data from each segment (Fig. 10*D*) showed that for animals in the male bladder experiment, there was a stronger weighting of afferent projections to the SDCom of L6 than S1. This was also seen in two out of three animals in the female bladder experiment. Conversely, in each of the two urethra experiments (female urethra, male urethra), two out of three rats showed a stronger weighting to S1 than L6. As such, the analysis of the group data for each of the four experiments failed to detect a significant difference between L6 and S1 weighting. This variation was also reflected in the 95% confidence intervals (Fig. 10*E*).

## Discussion

This study provides the first systematic anatomic analysis of directly visualized LUT afferents across the L5-S2 spinal cord in both male and female rats. Our multiscale approach defined the circuitry from the microscopic (single cells and axons) to macroscopic (pan-segmental) scales, enabling us to visualize features of afferent projections that would be difficult to deduce from individual sections. This revealed a core fundamental organization of the sensory neural circuitry common to both the bladder and urethra of male and female rats, which overall was remarkably similar in its targeting to specific spinal regions and within spinal segments. However, our data suggested that the lateral spinal nucleus receives input from bladder afferents but not urethra afferents. Further differences between the bladder and urethra afferents in the weighting of afferent projections to the SDCom of L6 and S1 cannot be excluded because of the interanimal variability of this parameter in the two urethra groups (male, female). Quantitation of afferents in the SPN and SDCom has provided weightings for the two primary projections of LUT afferents that will inform network analyses and computational modeling of these circuits. Our results complement recent ultrastructural analyses of bladder afferents in the spinal cord (Park et al., 2019).

Most previous preclinical rodent studies on neural control of the LUT have aggregated data from L6 and S1 spinal segments, assuming them to be functionally similar. However, our study revealed distinct anatomic features between these segments, as well as between rostral and caudal L6. This was demonstrated within motor and premotor (preganglionic) components, and by the specific weighting of LUT afferent projections to spinal cord regions and segments. Therefore, it is critical that future studies of lumbosacral regulation of the LUT, or changes in this regulation during disease or following injury, clearly target analyses or perturbations to specific spinal cord segments and subsegments.

Sensory activity driven by bladder distension is critical for initiating micturition that is mediated by preganglionic neurons of the L6-S1 cord. Sensory innervation of the proximal urethra has been studied much less than the bladder, but examples of its function include flow detection and contribution to the guarding reflex (Danziger and Grill, 2015, 2016). Our anatomic visualization of sensory innervation from the bladder and urethra demonstrated a strong association with a region adjacent to the SPN (primarily lateral Lamina V) and with the SPN itself. However, whereas LUT afferents project across the entire L6 segment, the preganglionic neurons exist only in the caudal half, raising new questions about afferent connectivity within the more rostral circuitry. One possibility is that local circuit interneurons regulating LUT function extend more rostrally than LUT-regulating preganglionic neurons. Although no chemoarchitectural markers have been identified to delineate this entire group of interneurons, a subpopulation near the SPN expresses TH (Hou et al., 2016a, 2021). In the current study, we found that TH neurons extend more rostrally than preganglionic neurons and we have previously reported that cystometry evokes c-Fos expression in this region and throughout the length of L6 (Wiedmann et al., 2021). However, we also note that only a minority of LUT afferents were closely associated with TH neurons. Therefore, development of new tools or molecular markers to map the entire local interneuron population relevant to LUT function will facilitate functional interpretation of this afferent innervation, in rostral L6 and throughout the lumbosacral cord.

Local circuit interneurons near the SPN may also be relevant to our observed extension of bladder and urethra afferents to L5 and S2 segments; although in lower density than L6 and S1, this projection was unexpected. Alternatively, it is possible that these sparse but broadly distributed afferents influence the regulatory circuits for sphincters, pelvic floor, and tail. The route by which LUT afferents reach these spinal segments should also be considered, as retrograde tracing studies have shown that projections from L3-L5 and S2 DRG are rare or absent (Keast and de Groat, 1992). Therefore, the LUT afferents we identified in L5 and S2 likely originate from L6 and S1 DRG, projecting within the cord to adjacent segments.

The primary trajectory of LUT afferents was the lateral collateral pathway, projecting to the SPN and the region immediately dorsal to the SPN, lateral Lamina V. Many LUT afferents within the SPN showed close appositions to somata or dendrites of preganglionic neurons (defined by ChAT immunoreactivity). In each animal, regardless of LUT region or sex, afferent innervation of the SPN was more heavily weighted to the S1 than the L6 segment. Limited information is available to further interpret this connectivity, as there is evidence both for and against direct synaptic communication between primary afferents and sacral preganglionic neurons (Mawe et al., 1984; Llewellyn-Smith et al., 2007; Persson and Havton, 2008). A recent elegant study of bladder afferent ultrastructure within the SPN of rats (Park et al., 2019) identified and characterized many synapses (e.g., on dendrites) but did not incorporate a method to confirm that the postsynaptic structures originated from preganglionic neurons. Also relevant to potentially direct afferent innervation of preganglionic neurons is the intercalated nucleus, which contains their long, medially-projecting dendrites that extend almost as far as the central canal (Nadelhaft and Booth, 1984). This region is also strongly innervated by Barrington’s nucleus (pontine micturition center; (Hou et al., 2016b; Verstegen et al., 2017). We identified bladder and urethra afferents strongly aggregated in the intercalated nucleus. However, close associations of afferents here and in the SPN will require electron microscopy or other direct measures of synaptic communication to infer function. If supported, this will contrast much of the literature on LUT reflex mechanisms that emphasizes circuits requiring spinal interneurons and supraspinal projections. Because local circuit interneurons are embedded within the SPN, future higher resolution studies of LUT afferents will require simultaneous definition of the preganglionic population (e.g., by ChAT immunoreactivity or retrograde tracing from the periphery).

The second major trajectory of LUT afferents in the spinal cord gray matter, the medial collateral pathway, innervates the SDCom (also previously identified as the dorsal commissural nucleus or dorsal gray commissure). We found that the afferent innervation of the SDCom projected not only across the full length of the L6 and S1 segments, but also extended into L5 and S2. In comparison to the SPN, afferent innervation of the SDCom was weighted differently across L6 and S1 segments, in many animals the bladder afferents projecting to the SDCom being more strongly weighted to L6 than S1. However, the higher level of intersubject variability of afferent innervation quantified in this region prevented further interpretation of this data. The SDCom is strongly innervated by Barrington’s nucleus (Ding et al., 1997; Hou et al., 2016b; Verstegen et al., 2017; Keller et al., 2018) and implicated in both bladder and urethra regulation. Continuous cystometry evokes immediate early gene expression in putative local interneurons within the SDCom (Cruz et al., 1994; Wiedmann et al., 2021), which could be initiated by both bladder and urethra afferents during the voiding cycle. This region also contains interneurons involved in urethral rhabdosphincter regulation, identified following transsynaptic tracing (Vizzard et al., 1995; Nadelhaft and Vera, 1996; Park et al., 1997). Additional roles of this region are indicated by immediate early gene mapping and transsynaptic labeling of motor pathways, which have identified responses to nociceptive stimuli and interneurons related to urogenital reflexes; these predominate in L6 (Honda, 1985; Birder and de Groat, 1992; Chinapen et al., 1992; Berkley et al., 1993; Vizzard, 2000; Marson et al., 2003; Marson and Murphy, 2006; Dobberfuhl et al., 2014). Together, these studies indicate that the SDCom is a site for potentially complex integration of afferent, local circuit spinal and descending supraspinal signaling pathways across many segments of spinal cord, and that the role of this circuitry in LUT function may differ between L6 and S1 segments.

An important aspect of our approach is that in visceral afferents CTB identifies all major populations of sensory neurons (Robertson et al., 1992; Wang et al., 1998), unlike the somatic afferent system where CTB preferentially labels the myelinated class (Robertson and Grant, 1989; Robertson et al., 1991). Electrophysiological and immunohistochemical studies have demonstrated specific features of myelinated (A-δ) and unmyelinated (C) classes of bladder and urethra afferents in rats (Yoshimura et al., 1998, 2003; Forrest et al., 2013; Danziger and Grill, 2016; Meerschaert et al., 2020), noting that the proportion considered to be myelinated varies across approaches and studies. We verified the uptake of CTB by both populations in our study, further showing that a major nociceptive subclass was labeled, identified by TRPV1 expression. We did not perform immunohistochemical characterization of LUT afferents within spinal cord regions but did observe a sparse but consistent innervation of superficial laminae in the dorsal horn, as expected for nociceptive projections and documented by electron microscopy (Park et al., 2019). This innervation was sparse in comparison to several other regions but is not the only site of LUT nociceptive signaling in the L6-S1 cord (Cruz et al., 1996; Park et al., 1997; Lagos and Ballejo, 2004). Moreover, many sensory projections in the medial collateral pathway of the L6-S1 cord express GFRα3, a feature of many TRPV1-positive nociceptors innervating the bladder (Forrest and Keast, 2008; Forrest et al., 2013). Although rare, we also identified bladder afferents within the lateral spinal nucleus, a site that is strongly implicated in nociceptive function (Jiang et al., 1999; Sikandar et al., 2017; Gutierrez-Mecinas et al., 2018). A logical extension of this study would be to separately quantify different classes of CTB-labeled LUT afferents in the spinal cord, e.g., to determine whether myelinated and unmyelinated afferents project differently to the SPN, SDCom or other gray matter regions, or project with different weightings to L6 and S1 segments.

Our study was focused on LUT afferents in specific regions of the spinal cord, but these same regions are also involved in regulation of the lower bowel and reproductive organs. A comparable study on afferents from these regions would greatly inform our understanding of pelvic visceral function and dysfunction, potentially demonstrating different patterning and strengths of projection to specific spinal cord levels and subregions. An application of our approach to map the afferent innervation of more distal regions of the urethra and the urethral rhabdosphincter would also be informative. In female rats our tracing occasionally identified small numbers of sphincter motor neurons because of tracer exposure at the rostral region of the sphincter during microinjection of the proximal urethra, but a targeted larger injection to this area is necessary to specifically identify its afferent innervation. It would also be valuable to use CTB tracing to map projections of LUT and other pelvic afferents to more rostral regions of the cord (lower thoracic and upper lumbar segments), where sympathetic spinal circuits reside and a separate population of visceral afferents project (Applebaum et al., 1980; de Groat et al., 2015). For the LUT, this is a smaller population of afferents compared with those projecting from L6-S1 ganglia (Vera and Nadelhaft, 1992).

In conclusion, new maps of bladder and urethra afferents have revealed heterogeneity within the lumbosacral cord itself, notably the L6 segment, and projection of bladder and urethra afferents to spinal levels beyond those that house preganglionic neurons. We found subtle or no detectable sex differences. This fundamental dataset and approach form a foundation for future studies to probe the impact on LUT afferent signaling of experimental perturbations, and changes during the life cycle.

## Synthesis

Reviewing Editor: Jeffery Twiss, University of South Carolina Decisions are customarily a result of the Reviewing Editor and the peer reviewers coming together and discussing their recommendations until a consensus is reached. When revisions are invited, a fact-based synthesis statement explaining their decision and outlining what is needed to prepare a revision will be listed below. The following reviewer(s) agreed to reveal their identity: Shaoping Hou.

Overall the reviewers and reviewing editor regard your manuscript as an incredibly detailed and careful analysis of afferent projection for two important LUT structures (bladder and urethra) that raises novel findings. In particular, you report that LUT afferents are not only present in rostral L6 but also project to spinal segments with no preganglionic neurons as well as the relationship between LUT afferents and TH+ neurons in the lumbosacral spinal cord. Reviewers also noted the quality of the work and the abundant technical information provided in methodology, which will be useful for future work. One reviewer noted that ‘this kind of data is critical for our understanding of the role of these afferent populations and what portions of the spinal cord are directly affected by their input’. Consequently, it could set the basis for both future basic science studies investigating how afferent input is integrated in the spinal cord to regulate organ function as well as for translational studies that seek to develop treatments for LUT dysfunction (e.g., spinal stimulators). Reviewers did raise several concerns that will need to be addressed before further considering this work for publication. These are outlined below as major and minor points:

Major points -

1. A primary concern is that there is a somewhat forced effort to find quantitative differences between the distribution of bladder and urethral afferents. This is reflected in part by questions on the validity of the statistics tests used (see point # 2). But a reviewer also raised the question of whether the findings are biologically relevant regardless of the appropriateness of the statistics used. In our online discussion, the concern was offered on interpreting those data given the variability in afferent inputs. We do not mean to imply that the results are not important, rather there was concern that trying to put a number to how urethral input is different from bladder input could lead to mis-interpretations of the biological relevance of the differences by readers and perpetuation of those in the literature. We request that you provide some level of discussion on biological relevance and clearly qualify your findings and conclusions with this in mind.

2. The determination of statistical significance is performed using a two-tailed t-test on the AUC for the projections to the spinal cord. Concerns were raised that using a t-test to look for significance between in the distribution of two types of afferents (bladder and urethra), in two sexes, across two different spinal segments. Please provide a clear rationale or provide alternate tests that incorporate multiple variables in the calculation.

3. Please add a brief discussion for what is known about the relative contribution of C-fiber and A-fibers to innervation of the bladder and urethra in rat if known. CTB labels both populations of visceral afferents. Could any of the potential differences in the projections to the SPN and SDCom be due to differences in these types of fibers?

4. LUT afferents mainly project to two distinct spinal segments in rats: T13/L1 and L6/S1. The former is related to sympathetic activity while the latter to parasympathetic control. This study only examined the lower entry level and the upper was ignored. Please give a rationale for restricting the work to lower entry level.

5. There is no clear boundary between adjacent spinal segments, such as L5 and L6. What kind of marker or criteria was used to distinguish them? This is important because the rostral or caudal part of the segment was described for afferent distribution. The information should be provided in the methods.

Minor concerns:

1. The 1st sentence in the Introduction, “......to ensure that autonomic control of the ......”.

2. The 1st sentence on page 4, remove parenthesis.

3. It seems CTB was injected into the bladder and urethra in different rats, but this information was unclear in the methods (page 5-6). Please clarify.
